# Supplementary material for: Critical evaluation of in situ analyses for the characterisation of red pigments in rock paintings: A case study from El Castillo, Spain
Source: PLoS One. 2022 Jan 24;17(1):e0262143. doi: 10.1371/journal.pone.0262143 (PMC8786193; doi:10.1371/journal.pone.0262143)
Supplement: S1 File — (PDF) [file pone.0262143.s001.pdf]

Critical evaluation of *in situ* analyses for the characterisation of red pigments in rock paintings: a case study from El Castillo, Spain

Laure Dayet*, Francesco d'Errico, Marcos García Diez, João Zilhão

*Corresponding author:
laure.dayet@gmail.com

Supplementary information

Location of pXRF analyses

Fig SI-1. Corridor of the disks, part 1 / Galeria de los discos.

Fig SI-2. Corridor of the disks, part 2 / Galeria de los discos.

Fig SI-3. Corridor of the disks, part 3 / Galeria de los discos.

Fig SI-4. Panel of the hands / Techo de las manos.

Fig SI-5. Panel of the polychromes / Panel de los polychromos.

Fig SI-6. Cave wall

LOCATION OF pXRF ANALYSES

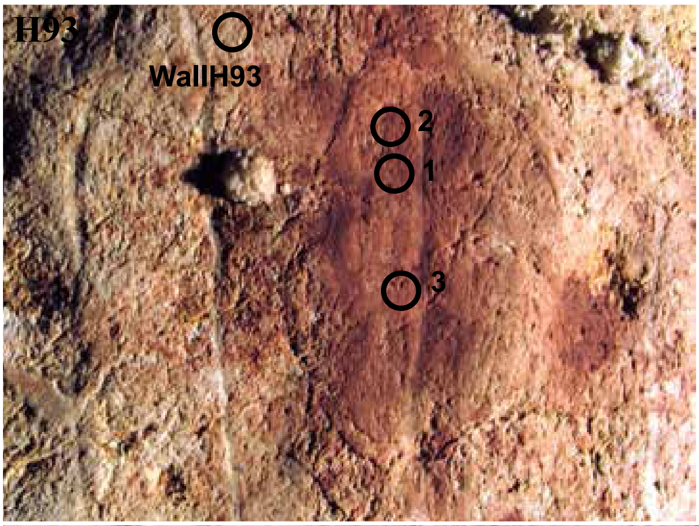

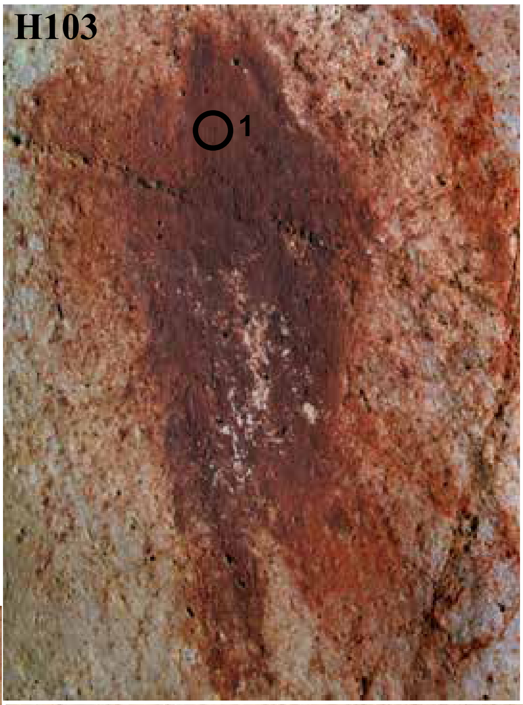

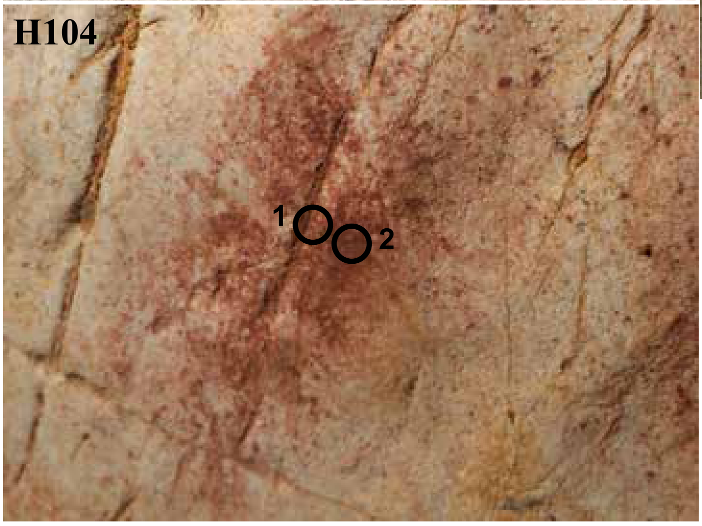

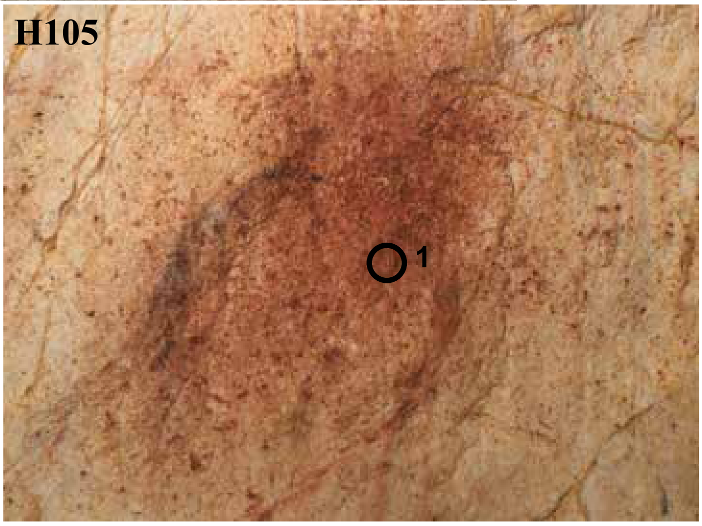

Figure SI-1 - Corridor of the disks, part 1 / Galeria de los discos

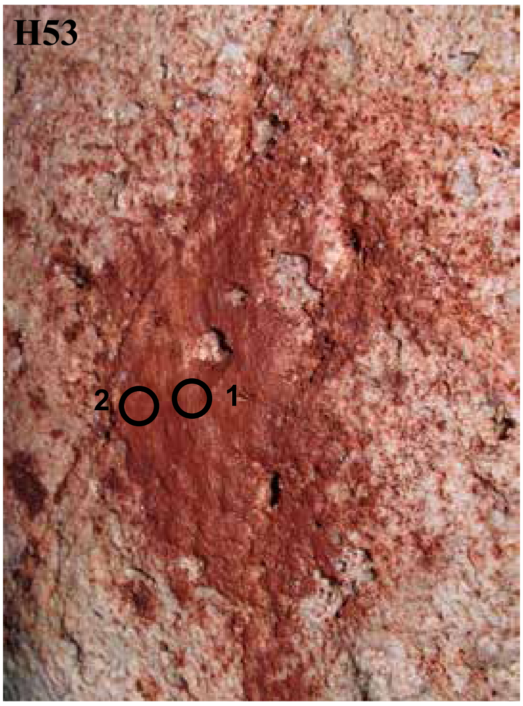

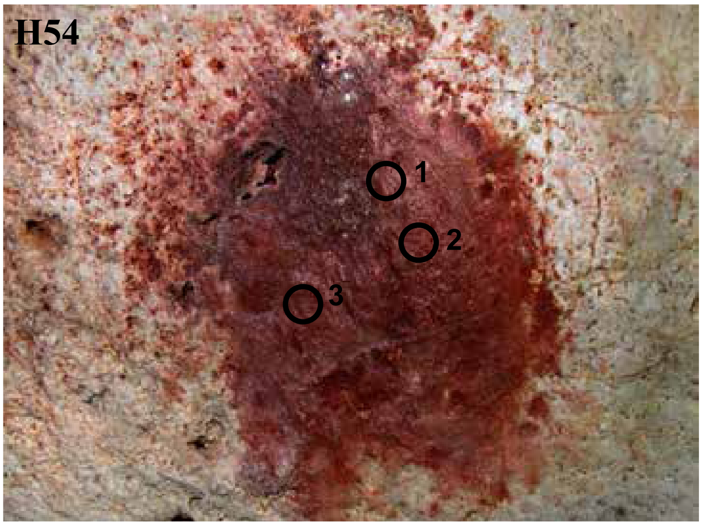

Figure SI-1 - Corridor of the disks, part 2 / Galeria de los discos

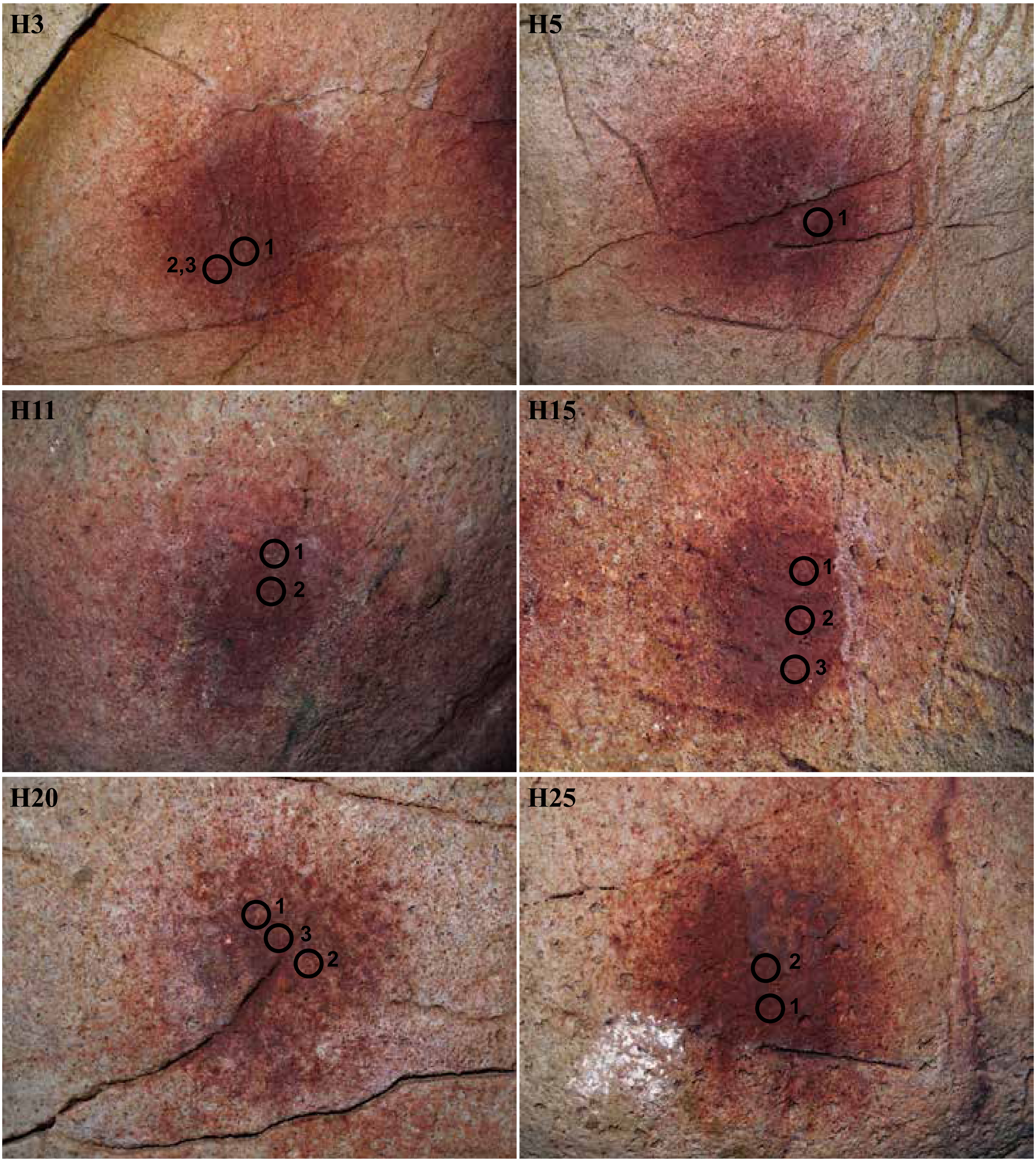

Figure SI-3 - Corridor of the disks, part 3 / Galeria de los discos

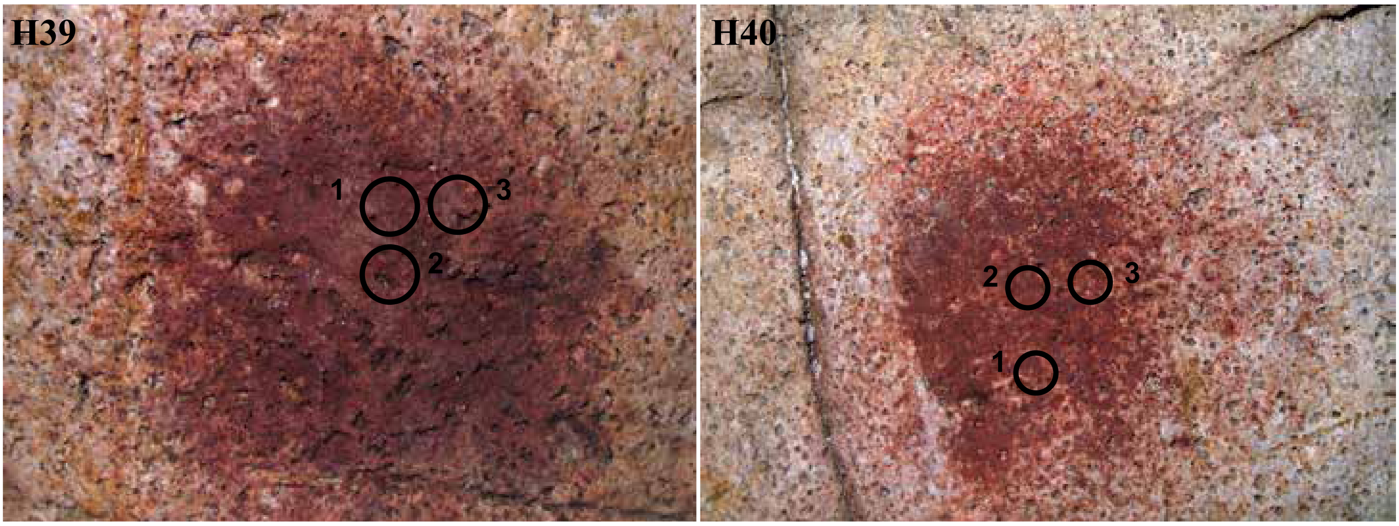

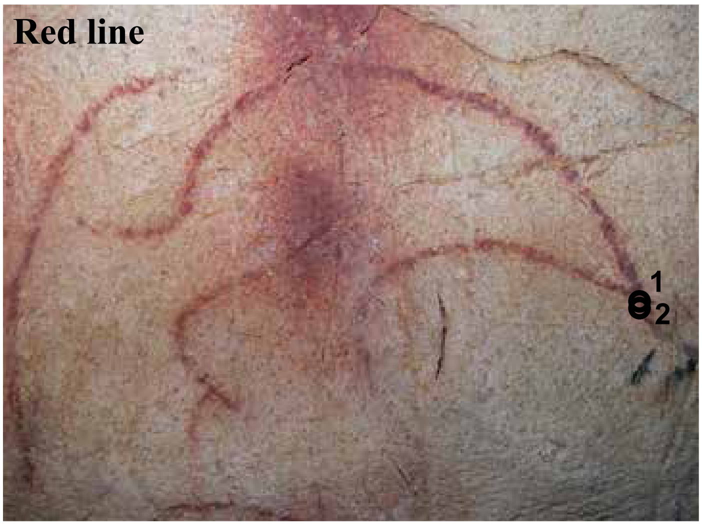

Figure SI-3 continuation - Corridor of the disks, part 3 / Galeria de los discos

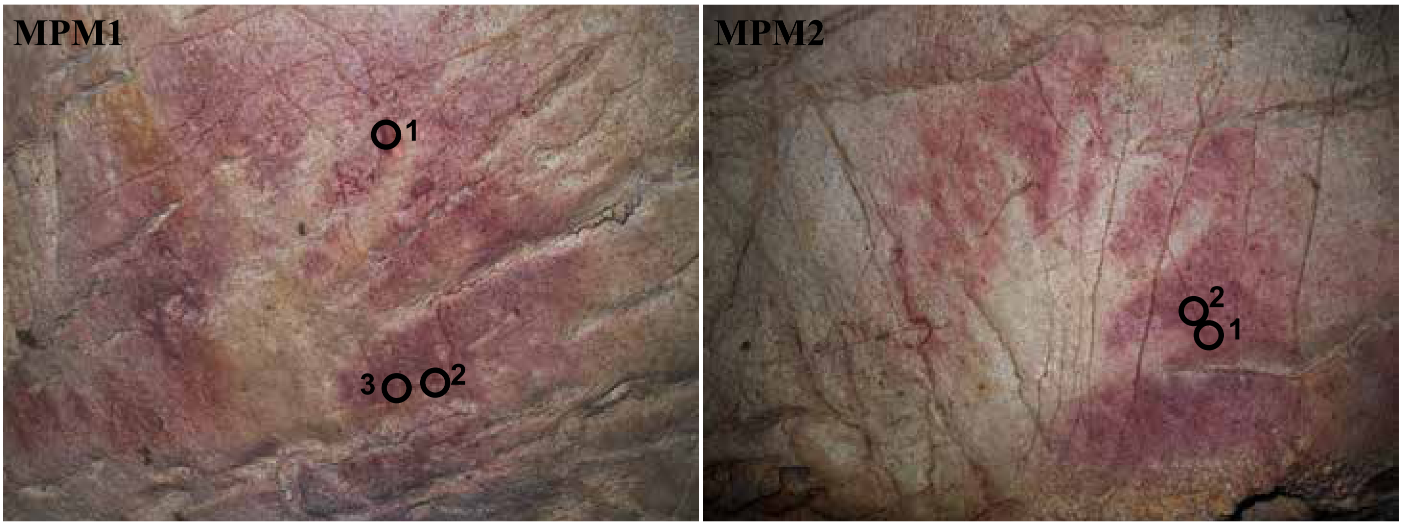

Figure SI-4 - Panel of the hands / Techo de las manos

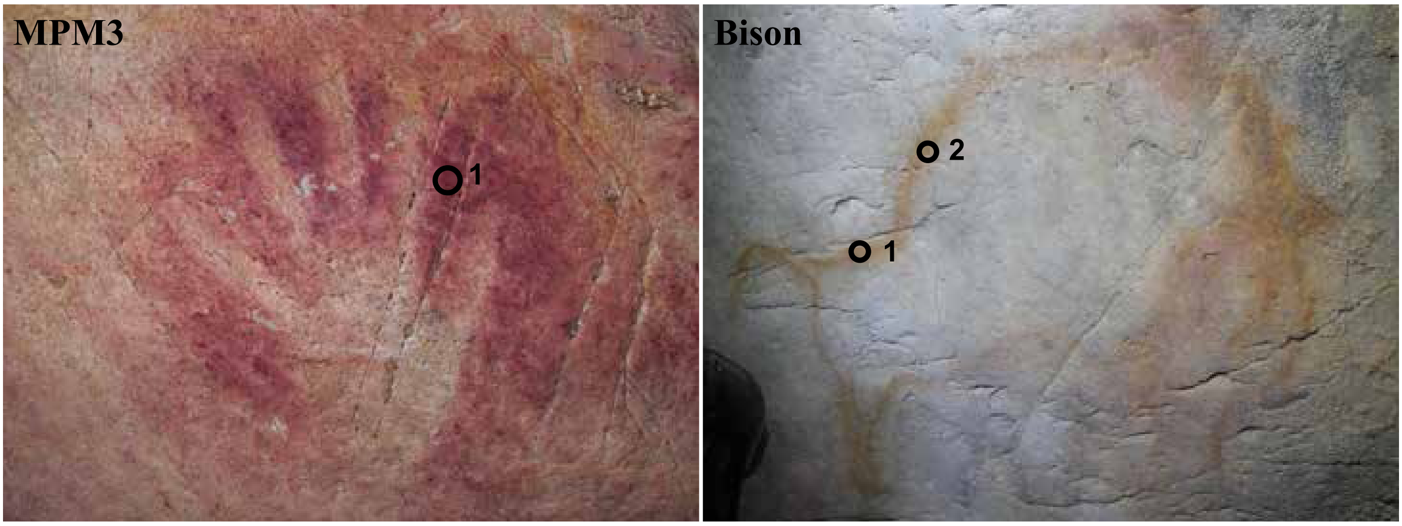

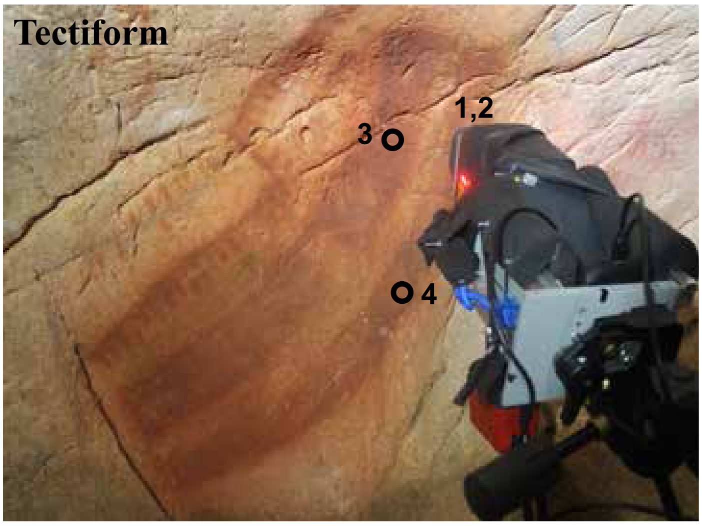

Figure SI-4 continuation - Panel of the hands / Techo de las manos

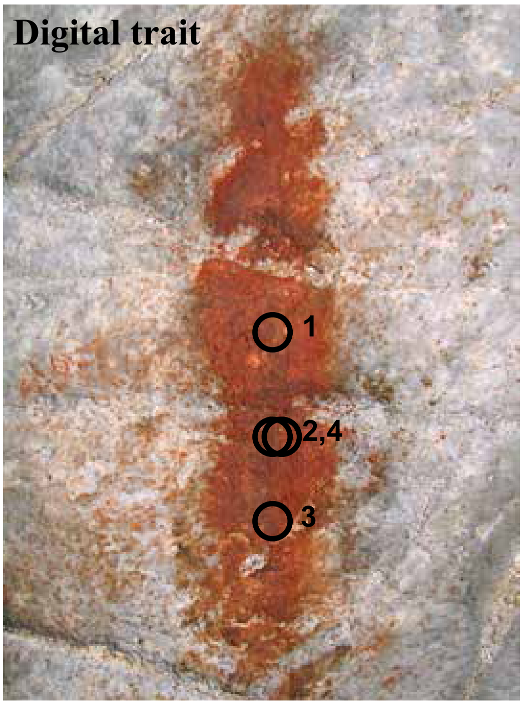

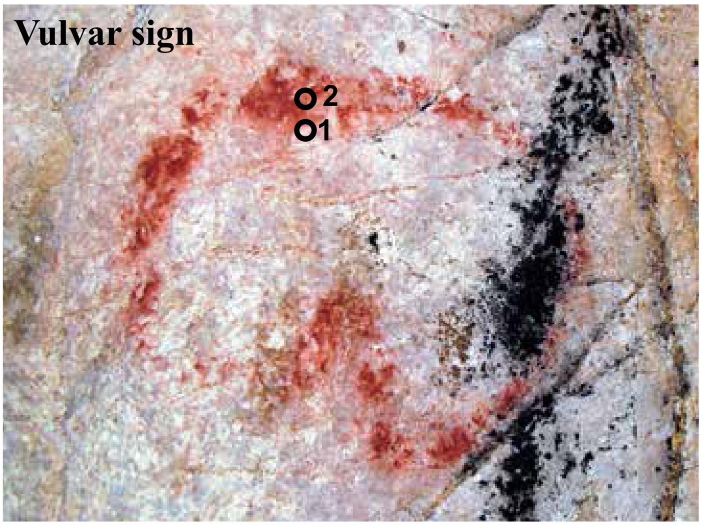

Figure SI-5 - Panel of the polychromes / Panel de los polychromos

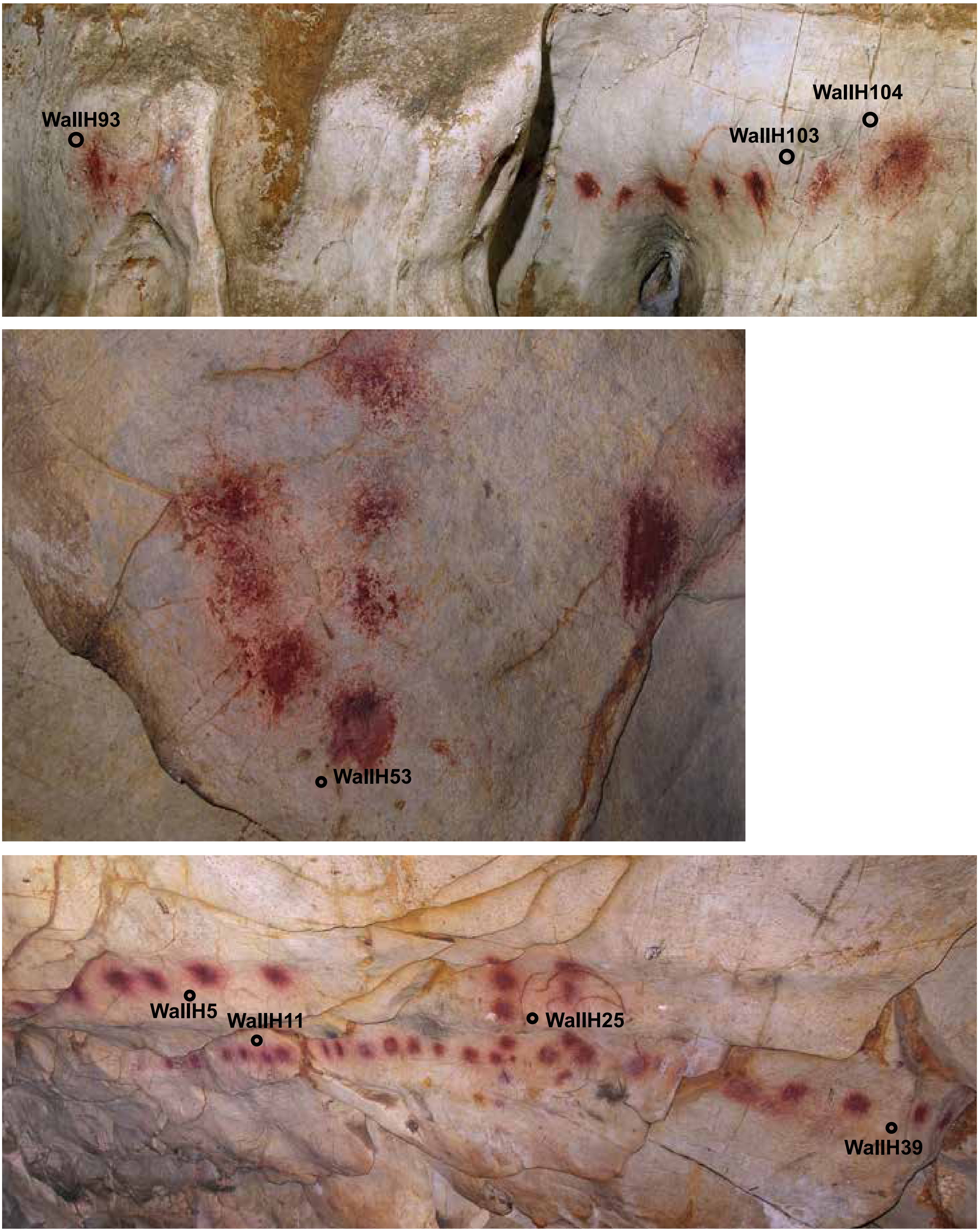

Figure SI-6 - Cave wall
