# Supplementary material for: Critical evaluation of in situ analyses for the characterisation of red pigments in rock paintings: A case study from El Castillo, Spain
Source: PLoS One. 2022 Jan 24;17(1):e0262143. doi: 10.1371/journal.pone.0262143 (PMC8786193; doi:10.1371/journal.pone.0262143)
Supplement: Attachment — Submitted filename: Review for dayet et al.docx [file pone.0262143.s003.docx]

# Review for « Critical evaluation of in situ analyses for the characterisation of red pigments in rock paintings: a case study from El Castillo, Spain », Dayet et al.

Dear colleagues,

It was a pleasure to read this article by Dayet et al. on their p-XRF analysis at the El Castillo cave. This article is well-written, easy to follow and present a very important contribution to the use of p-XRF for rock art analysis. The processing of the data with different methods with the aim to understand biases that may be introduced by this method is very welcome at a time when such a method is now widely used without the required cares. Although focusing on the rock art case, the results presented here are relevant for the study of archaeological materials with p-XRF in general.

All data acquired through this study are available in supplementary materials, which allow any researcher to reproduce the processing. I thank the authors for this.

In conclusion, I recommend this article to be accepted with minor revisions listed below.

## Major comments

Lines 97 to 116: This paragraph is very interesting and reflects the choices that are constrained by the use of raw materials and technics of applications. However, I would emphasis that a consideration is missing here: the type of binders chosen. Indeed, your binder has to be chosen in adequation to your raw materials, its texture and granulometry, and to the application technique. Although you mention the paint materials at large, I feel it may be needed to emphasise that both the colouring materials and the binders are of importance. I would also suggest elaborating a little more on “technical facts” (predictable gestures) and “degrees of facts” (unpredictable gestures linked to social behaviours): distinctions from Leroi-Gourhan’s theory on the “chaîne opératoire”. Indeed, the materials chosen, their preparation and the application techniques may be related to specific spiritualities or rituals and gestures adapted to fit the use of such materials/techniques.

Lines 118 to 137: The changes in the raw materials/paint recipes may also be related to trade networks and to exchanges of believes/behaviours. We may also consider that several groups with different behaviours came to paint in the same cave at the same “period”. The dating methods do not allow a very precise chronology for such very old periods, with nomadic tribes moving in large areas. Hence, different paint recipes may only be related to several tribes painting in the same cave with different techniques.

Although you were not able to analyse the same figures that d’Errico et al. 2016, I feel like links between the two are missing. A table may be added that summarize similarities and differences between your study and d’Errico et al. 2016.

You included a yellow paint as part of your study but you stated several times that you will focus on the red paintings (line 79 for example). But at the end, you mention that you used the yellow painted figure to determine if it is distinguishable from the red paintings. I feel that this information should come at the beginning of the article and you may also state why you chose only one yellow paint: accessibility, thickness of the layer, chronology, other reason? This yellow painting is also not visible in your PCA figures. Was it considered? If so, I would suggest marking it with a different bullet in your figures. It will help the reader.

At lines 645-646, you quickly mention the variations within one figure but it is not visible in the rest of your paper and in your figures. I feel it would be useful to have more information regarding the reproducibility of the measures on one single figure to better appreciate the issues of differentiation between several figures. I would suggest adding a quick paragraph on this and maybe emphasising them for a couple of relevant examples in the figures.

I would like to emphasis a few more references for your study (for p-XRF analysis, and for the recognition of paint pots and their chronological implications):

- MacDonald, B. L., et al. (2019). "Hunter-Gatherers Harvested and Heated Microbial Biogenic Iron Oxides to Produce Rock Art Pigment." Scientific Reports 9(1): 1-13.
- MacDonald, B. L. (2015). Methodological developments for the geochemical analysis of ochre from archaeological contexts: Case studies from British Columbia and Ontario, Canada. Anthropology, McMaster University. PhD: 209.
- Trosseau, A., et al. (2021). "In-situ XRF study of black colouring matter of the Palaeolithic figures in the Font-de-Gaume cave." Journal of Analytical Atomic Spectrometry.
- Castañeda, A. M., et al. (2019). "Portable X-ray fluorescence of Lower Pecos painted pebbles: New insights regarding pigment choice and chronology." Journal of Archaeological Science: Reports 25: 56-71.
- Bonneau, A., et al. (2021). "Characterization and dating of San rock art in the Metolong catchment, Lesotho: A preliminary investigation of technological and stylistic changes." Quaternary International.
- Bonneau, A., et al. (2017). "A pigment characterization approach to selection of dating methods and interpretation of rock art: the case of the Mikinak site, Lake Wapizagonke, Quebec, Canada." Archaeometry 59(5): 834-851
- Sepúlveda, M. (2021). "Making visible the invisible. A microarchaeology approach and an Archaeology of Color perspective for rock art paintings from the southern cone of South America." Quaternary International 572: 5-23.

## Minor comments

Text:

- There are several lists of references for which I would suggest adding *e.g.* at the beginning. Although your review of the literature is very good, I was able to spot some more references (see above). To avoid very long reference lists, here are the lines with the reference list to which I would add *e.g.* at the beginning:
  - Line 31
  - Line 92
  - Line 147
- Lines 66 – 67: I would suggest presenting the U-series dates as follow, which is a more conventional way to report the age:
  - 41.4±0.6 ka
  - 35.7±0.6 ka
- Line 163 – 175: on the use of *in-situ* Raman spectroscopy, I would suggest emphasising the possibility of burnt marks if the system is not used correctly. Although, the instruments are getting safer and the spot sizes are getting smaller, Raman spectroscopy remains a “risk of damage” if not used correctly.
- Line 180 – 181: amorphous carbon cannot be “identified” with p-XRF, it can be assumed if no Mn or Fe is present in the spectrum. It is indeed hair-splitting but this sentence may give false information to non-initiate readers.
- Line 330: you mentioned a red natural deposit in Sala de los Polícromos that you analysed. Is there any photo related to this natural deposit? How is it called in your data set?
- Line 363: “paint pots” rather than “pot paints”.
- Lines 707 – 711: I would suggest removing the references in your points. This conclusion presents the results of your study, which indeed confirm previous ones. However, as it is written, it looks like these conclusions are drawn from previous studies and not from this paper. You may emphasis more your own contribution to these previous studies. This paper is a really important one with the several ways of processing data and their critical evaluation.

Tables and figures:

- Table 2: I would suggest aligning numbers of “p-XRF analyses” and “microscope” columns in the centre. It will make it more understandable.
- Table 3 caption: I would suggest repeating the article in which the compositional data are extracted for Ref-Fe1.
- Fig 10: The lines are difficult to understand and “hide” a part of the graphic. I would suggest adding a table on the right of the graphic to list the correlations between the paintings and the wall analyses.
